# Students' impression management styles and school adjustment: the mediating role of self-esteem and the moderating role of age

**DOI:** 10.3389/fpsyg.2025.1550213

**Published:** 2025-08-29

**Authors:** Xiaoqian Tang, Tinghu Kang, Yalin Liu, Nan Li

**Affiliations:** ^1^School of Finance and Economics, Jinan Vocational College, Jinan, China; ^2^School of Psychology, Northwest Normal University, Lanzhou, China; ^3^School of Educational Sciences, Northwest Normal University, Lanzhou, China

**Keywords:** impression management, impression management style, school adjustment, self-esteem, age

## Abstract

School adjustment plays a critical role in students' development by fostering self-confidence, social competence, effective study habits, and smoother integration into the academic community. Positive adjustment not only promotes physical and mental wellbeing but also enhances learning efficiency and lays a strong foundation for future academic and career success. However, not all college students adapt smoothly to campus life. Variations in interpersonal communication styles, developmental backgrounds, and self-esteem levels can lead to differences in school adjustment. This study examined the relationship between impression management (IM) styles and school adjustment among college students. Data were collected from 682 students across four universities in China. The results showed that authentic acting IM style was positively associated with school adjustment, while role acting IM style was negatively associated. Moreover, self-esteem mediated the relationship between IM styles and school adjustment. Age was also found to moderate the link between authentic acting IM and self-esteem.

## Introduction

The university stage represents a critical period of individual development, serving as a transitional phase in physical, psychological, and cognitive domains. It also functions as a preparatory stage for individuals to fully enter society and assume independent social responsibilities, making it the second major developmental period in life (Raza et al., 2021). During this time, individuals undergo significant environmental changes—leaving their parents and original social networks—which requires them to independently navigate and reconstruct interpersonal relationships, while acquiring essential life skills (Renk and Smith, 2007). Concurrently, they must address developmental tasks such as establishing a stable sense of self in terms of personality and social identity (Seidman and French, 2004). Due to the shifts in living and academic environments, many university students experience maladjustment, often reflected in confusion or distress related to interpersonal relationships, academic or career development, and emotional regulation (Arnett, 2016). These challenges can heighten the risk of psychological problems, making adjustment difficulties particularly salient during this period (Hamre and Pianta, 2001). Therefore, facilitating students' rapid adaptation to their new environment, fostering social support systems, and promoting overall adjustment to university life are essential—not only for their current functioning but also for their long-term personal and professional development.

### Impression management

Impression management (IM) focuses on people's behaviors in order to create and maintain an ideal image of themselves and thus change others' views (Gardner and Martinko's, 1998). It may be to create an ideal new image, or to protect and maintain an existing image (Bolino et al., 2006). Marcus (2009) categorized IM into “honest self-representation” and “dishonest self-representation”. In the best case, people will remain completely honest, which is called honest self-presentation, however, there are also people who consciously deceive and falsify in order to leave a good impression on others, which is dishonest self-expression (Uziel, 2014). In the field of organizational behavior, IM refers to the process by which individuals attempt to control or shape others' perceptions of them. This process often involves strategies aimed at influencing the way one is viewed by others, particularly in a professional setting. The goal is often to create a favorable impression to achieve personal or organizational objectives (Bolino et al., 2006).

A school is not only an educational institution where students acquire cultural knowledge but also a structured social organization. Within this context, impression management (IM) plays a crucial role. IM is relevant not only to individual students but also to the cultivation of a harmonious interpersonal climate on campus. In today's competitive environment, college students often consciously or unconsciously engage in IM to shape how others perceive them (Bolino et al., 2016). Safay (2010) identified two primary IM styles based on interview analysis: authentic acting and role acting. These styles reflect general behavioral tendencies aimed at influencing others' impressions. Authentic acting IM refers to efforts to present oneself in a truthful yet socially appropriate manner. Individuals employing this style strive to create positive impressions by sharing personal experiences, emotions, needs, and beliefs that align with their true selves, while also considering the situational context, including their goals and the audience's expectations (Harter, 2002; Schlenker and Weigold, 1992). In contrast, those who adopt a role acting IM style focus on meeting others' expectations, even if it requires presenting an image that is inconsistent with their actual self (Leary, 1995). This often involves projecting socially desirable traits, concealing genuine emotions, or pretending to have qualities they do not possess in order to gain approval or acceptance. It should be noted that the present study adopts this typology to describe behavioral tendencies and adjustment strategies in impression management, rather than to make moral judgments about either the “authentic” or “role-based” style. This classification differs from the traditional distinction between “honest” and “deceptive” self-presentation in impression management research, which focuses on the consistency between presented information and one's inner reality (Schlenker and Weigold, 1992). In contrast, the styles discussed here reflect individuals' preferences and habitual approaches to self-presentation in social interactions.

### School adjustment and impression management style

School adjustment is a multidimensional concept, and there is currently no unified concept in the field of psychology. Some scholars believe that the concept of school adjustment goes beyond students' simple academic achievements and also covers their emotional responses and attitudes toward school, as well as their participation and involvement in the school environment (Ladd et al., 1997). Some scholars believe that school adjustment is a condition in which students are happily engaged in the school environment and achieve academic success in the school context, which can be measured by three indicators including emotion, study and interpersonal relationship. Learning adjustment, interpersonal adjustment and emotional adjustment should be the core aspects of school adjustment (Zeng and Zhang, 2010). It has been pointed out that university students should strive to have a high level of school adjustment because it not only improves their academic performance, physical and mental health, personality refinement, and social functioning during their university years, but also enhances their self-confidence, as well as being related to their future social adjustment and professional career (Perera and McIlveen, 2014).

Previous studies have found that variables related to external environmental conditions such as students' academic achievement, peer relationships, and teacher-student relationships can predict their adjustment in school (Chen et al., 1992). Students with high self-esteem are less likely to have social anxiety and school adjustment problems, and have higher life satisfaction and happiness (Du et al., 2022). Students with strong self-control can better manage their behavior, establish positive interpersonal relationships, and effectively cope with academic and social pressures, so as to better adapt to school (Hong and Cui, 2020; Duckworth et al., 2019). In summary, there are many factors that affect school adjustment. Internal factors include age, self-esteem, self-control, personality traits, etc. (Aldrup et al., 2018), and external factors include family education methods, social support, etc.

As mentioned earlier, the university stage is an important stage for students to leave their parents, face relationships on their own and adjust to a new campus life. One study found that university students' self-management promotes school adjustment (Jing and Kim, 2021). Students can improve their adjustment to the school environment through positive self-management tools such as emotional regulation, mindfulness, and self-control (Pintrich, 2004). Positive self-management contributes to the development of essential skills in areas such as academics, social interaction, emotional regulation, and stress management, enabling students to better adjust to school life. Conversely, negative self-management may create obstacles in these areas, affecting students' school adjustment. Self-efficacy, social skills, and self-concept all mediate the relationship between self-management and school adjustment (Li, 2019).

Impression management (IM), as a form of self-regulation, refers to strategies individuals use to influence how others perceive them (Leary and Kowalski, 1990). Different IM styles can significantly impact university students' interpersonal relationships, academic performance, mental health, and sense of belonging. Among these, interpersonal relationships are central to students' adaptation in the university setting. IM strategies can shape how others—peers or teachers—perceive and respond to students (Schlenker and Weigold, 1992). In academic environments where social interactions are frequent, students often employ various IM tactics to influence impressions formed by others (Hafiz et al., 2020). These impressions, in turn, can affect students' academic and social outcomes.

Empirical studies show that students use IM with teachers to gain favorable evaluations, including better grades. Authentic acting IM—aligned with one's true self—is positively associated with academic performance, whereas role acting IM—aimed at fulfilling perceived expectations—is negatively related (Hafiz et al., 2020). Moreover, authentic acting contributes to emotional stability and mental health by reducing the stress caused by internal-external inconsistencies. In contrast, role acting often increases psychological burden, fatigue, and emotional dysregulation, thereby heightening adjustment stress over time (Baumeister and Leary, 1995). Furthermore, authentic acting enhances social support and campus belonging by fostering genuine connections and acceptance from others, which helps students navigate academic and personal challenges (Deci and Ryan, 2000).

### The role of self-esteem in impression management and school adjustment

The concept of self-esteem was introduced by Rosenberg in 1965, who recognized self-esteem as an individual's attitudinal experience of the self, an emotional evaluation of the self on a negative or positive level. Driven by this need to enhance positive self-expression, individuals may exhibit positive behaviors, positive emotions, which can contribute to their job performance, interpersonal interactions, etc. (Terjesen et al., 2004).

Research has shown that self-esteem and positive coping styles are significantly positively associated with various dimensions of school adjustment, while self-esteem and negative coping styles are significantly negatively associated. Moreover, self-esteem serves as an important mediating variable between coping and school adjustment (Li, 2019). IM is closely linked to self-esteem among university students, as individuals often engage in IM to seek affirmation and maintain a positive self-image. When these self-related needs are met and individuals receive recognition from others, they are more likely to perceive and affirm positive aspects of themselves, thereby enhancing self-esteem (Chng et al., 2015). Different IM strategies lead to different social evaluations, which in turn affect individuals' levels of self-esteem.

On the other hand, self-esteem is one of the intra-individual factors of students' school adjustment (Aldrup et al., 2018), and it is an evaluation made and usually held by individuals about themselves. People with high self-esteem may have higher ambitions than people with low self-esteem, and in the face of failure, they are more willing to work for change than to attribute failure to incompetence and self-doubt. High self-esteem fosters confidence and determination to resolve difficulties and enables satisfaction from progress and success, so students with high self-esteem tend to have a good adjustment during the school adjustment process. When individuals have high levels of self-esteem, they tend to accept and identify with themselves, have high mental toughness and low sensitivity to external stimuli, and therefore show high adaptability to the environment, which implies that they have excellent school adjustment (Yoo and Lee, 2013).

### The role of age in self-esteem, impression management, and school adjustment

The moderating effect of age on self-esteem is reflected in changes at different stages of the life cycle. The results of a meta-analysis showed that self-esteem levels increased from 4 to 11 years old, remained stable from 11 to 15 years old, and increased strongly until the age of 30. This suggests that self-esteem improves with age during childhood and adolescence. However, the growth of self-esteem is not linear. It shows different characteristics and trends at different ages. This change is not only related to physiological decline, the transformation of social roles, and the accumulation of life experience, but also affected by social expectations and cultural background (Hauke and Abele, 2020). As they age, individuals may experience ups and downs in self-esteem, but the overall trend may be that self-esteem levels will gradually increase with the accumulation of life experience and the deepening of self-awareness (Gebauer et al., 2013).

Research shows that IM strategies vary among individuals of different ages. Young people usually rely on external feedback and present themselves by catering to and pleasing others, while as they age, their IM strategies tend to be more mature and introspective, relying more on the display of self-identity and professional ability (Jones and Pittman, 1982), especially older individuals who gain respect and social recognition by sharing wisdom and experience (Steverink et al., 2001). For college students, junior college students are faced with new learning tasks, interpersonal relationships and living environment, and they have to actively integrate into the university interpersonal communication environment, while senior college students are about to leave the university campus and begin to explore new career tasks such as job searching or further study. It means that the interpersonal communication context of college students of different ages has changed with the change of grade, and the impression management strategies they apply may also change accordingly.

In addition, a study explored the relationship between students' perceived social support in school and school happiness and found that age played a moderating role in this relationship. This suggests that students of different ages may have different feelings and reactions to school support, which in turn affects their school adjustment (Liu et al., 2016). Furthermore, Sun and Kang (2020) argued that at different stages of career development do not have the same IM style during interpersonal interactions. At the beginning of the career adjustment stage, individuals are in the exploratory stage and are more inclined to false IM in order to adapt to social expectations or integrate into a certain group as soon as possible; at the mature stage of career development, as they grow older and their experience and knowledge increase, individuals may increase their true IM. Thus, the impact of IM styles on university students' interpersonal interactions, and even academic performance, may be moderated by university students' different age stages.

Although students' IM style is related to self-esteem, which is one of the factors of school adjustment, no study has yet explored and examined the relationship and interaction mechanism between IM style and school adjustment by using self-esteem as a mediating variable. In view of this, the present study attempts to analysis the existence of a relationship between self-esteem between IM style and school adjustment, and explore the role of age in it. Therefore, this study proposes the following hypothesis:

**Hypothesis 1:** Students' authentic acting IM styles is positively related to their school adjustment, whereas role acting IM style is negatively related to their school adjustment, this relationship may also be moderated by the age stage of the university student.

**Hypothesis 2:** Self esteem plays a mediating role between IM style and school adjustment, and it can change depending on the age stage of the university student.

## Research methods

### Participants

Data for the study were collected online from June 15, 2023 to June 27, 2023 after obtaining permission from the Ethics Committee. In the online form, participants were informed about the study before they started answering the questions and answered the scale based on consent. This study adopted a convenience sampling method to recruit university student participants from four comprehensive universities located in the eastern, southern, western, and northwestern regions of China. The use of this method was primarily based on practical considerations related to research resources and data accessibility. Given the logistical constraints of conducting a large-scale, multi-regional survey, convenience sampling provided an efficient way to obtain valid samples across different regions. A total of 696 questionnaires were distributed in this study, and 682 valid data were retrieved due to partial completion. Among them, 294 (43%) were male students and 388 (57%) were female students. The age of the students ranged from 18–24 years with a mean age of (20.27 ± 1.51) years. The difference between the scores of the school adjustment scale for male students (*M* = 107.21; *SD* = 22.93) and the scores of the school adjustment scale for female students (*M* = 105.86; *SD* = 20.29) was not significant, *t* = 0.81; *p* = 0.42.

### Data collection tools

(1) Impression Management Styles Scale. The Maastricht (Safay, 2010) Impression Management Styles Scale (MIMSS) was used to measure IM style, e.g., “I don't pretend to be someone else at work.” The scale consists of 15 questions, with the first 7 questions measuring the IM style of the students‘ authentic acting style, and the last 8 questions measuring the IM style of the students' role acting style, and all items are scored on a 5-point scale ranging from 1 (completely disagree) to 5 (completely agree). The Cronbach's alpha coefficient for this scale was 0.78 for authentic acting and 0.8 for role acting style. The Cronbach's alpha coefficient in this study was 0.83 for authentic acting style and 0.79 for role acting style. It shows a good level of reliability and good stability of the scale.
(2) Self-Esteem Scale. Self-esteem was measured using the Self-Esteem Scale (SES) developed by Rosenberg (1965), e.g., “I feel that I have many good qualities.” The scale consists of 10 questions, 5 questions are reverse scored on a 4-point scale from 1 (very poorly) to 4 (very well). The Cronbach's alpha coefficient for this scale was 0.85, and in this study it was 0.85. It shows a good level of reliability and good stability of the scale.
(3) School adjustment Questionnaire. The School adjustment Questionnaire for University Students developed by Zeng and Zhang (2010) was used, which contains three aspects of academic, interpersonal, and physical and mental adjustment, for example, “I have a lot of friends at school.” The scale consists of 25 questions, 13 of which are reverse scored on a 6-point scale ranging from 1 (strongly disagree) to 6 (strongly agree). The internal Cronbach's alpha coefficient for this scale is 0.90, and the Cronbach's alpha coefficient for this study is 0.94. It shows a good level of reliability and good stability of the scale.

### Research procedures

All questionnaires were collected using group testing and collected on the spot, and after excluding invalid questionnaires, SPSS 25 was used for data analysis. The kurtosis and skewness values were checked for normality of the scale scores, and if these values are between ±1, the score distribution is normal. The Pearson correlation method was used for the relationship between the scale subdimensions, the Hayes macro method model 4 was used for the mediation analysis, and the Hayes macro method model 7 was used for the moderation analysis. In order to evaluate the fitting degree of the model, this study used various indicators, including r2, F, t, etc., to conduct statistical analysis and examine the significance level of *p* < 0.05 (Edwards and Lambert, 2007). In this case, to facilitate the interpretation of the results, the independent variables, mediating variables, dependent variables, and moderator variables were standardized before conducting hypothesis testing.

## Results and analysis

### Comparison of school adjustment among students of different genders and ages

Dividing students according to the stage of their growth and change during their university years, this paper divides the age into 2 levels, the low age (18–20 years old), and the high age (21–24 years old). The difference in scores between the high age school adjustment scale (*M* = 106.14; *SD* = 20.58) and the low age school adjustment Scale (*M* = 106.91; *SD* = 22.80) was not significant, *t* = 0.46; *p* = 0.66.

### Common method deviation test

In order to ensure the validity of the statistical analysis, the common method bias test was first conducted using SPSS, and the analysis showed that there were 9 factors (more than 1) with eigenroots greater than 1, and the variance explained by the largest factor was 27.637% (less than 40%), so there was no serious common method bias in this study (Aguirre-Urreta and Hu, 2019).

### Correlation analysis

As can be seen from Table 1, the correlation analysis of authentic acting IM style, role acting IM style, self-esteem and school adjustment found that there was a significant correlation among the variables. The correlation results provided preliminary support for our hypothesis that authentic acting IM style was positively correlated with school adjustment (*r* = 0.279, *p* < 0.01), and role acting IM style was negatively correlated with school adjustment (*r* = −0.085, *p* < 0.05). Hypothesis 1 was partially supported.

**Table 1:** Correlation analysis of all observed variables.

**Variable**	***M***	***SD***	**1**	**2**	**3**	**4**
1 Authentic acting style	25.450	4.903	1			
2 Role acting style	26.905	5.182	0.335^**^	1		
3 Self-Esteem	29.330	4.564	0.173^**^	−0.096^*^	1	
4 School adjustment	106.440	21.464	0.279^**^	−0.085^*^	0.624^**^	1

^*^P < 0.05, ^**^P < 0.01.

In addition, a multi-group analysis was conducted on the two dimensions of the impression management style scale, the authentic acting IM style and the role acting IM style. The paired sample T test results showed that there were differences between the two styles (*t* = −6.526, *p* < 0.001). Therefore, the two different IM styles will be divided into two variables for discussion below.

### The mediating role of self-esteem and the moderating role of age in impression management style and school adjustment

As shown in Table 1, the correlations between self-esteem and different IM styles and school adjustment all reached significant levels and continued to the next step of the analysis. Using different IM styles as the independent variable, school adjustment as the dependent variable, and self-esteem as the mediating variable, the macro PROCESS v4 model 4 in SPSS 25.0 was used to test for the presence of a mediating role in the relationship between different IM styles and school adjustment (Hayes, 2017).

#### The mediating role of self-esteem and the moderating role of age in the authentic acting IM style and school adjustment

**Figure 1:**
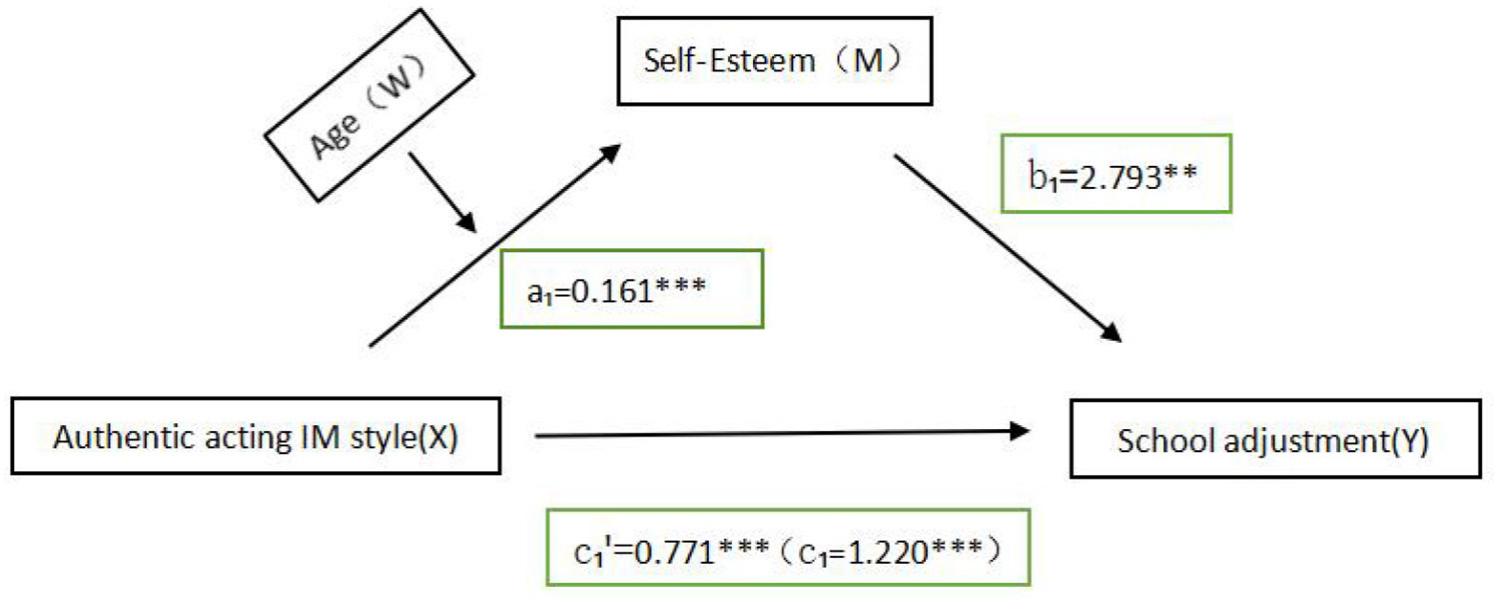
Conceptual model illustrating relationships between variables: Age (W) influences Self-Esteem (M) and Authentic Acting IM Style (X). Self-Esteem has a positive effect on School Adjustment (Y) with a beta value of 2.793. Authentic Acting IM Style directly influences School Adjustment, with a coefficient of 0.771. Paths show relationships with coefficients: a1 = 0.161 and c1 = 1.220. The mediating role of self-esteem in the authentic acting IM style and school adjustment, and the moderating role of age. ^**^P < 0.01, ^***^P < 0.001.

From Table 2, in the model, *r*^2^ is 0.648, indicating that the model fits. As derived from Figure 1, the regression equation for the path from authentic acting IM style to self-esteem was:


$$\begin{array}{c} \text{M}=25.237+0.161\text{X}, \end{array}$$

**Table 2:** Intermediary effect detection, decomposition table of total, direct, and indirect effects of self-esteem inauthentic acting IM styles and school adjustment.

**Regression equation**		**Overall fit index**		**Significance of regression coefficients**	
**Outcome Variables**	**Predictor variables**	***R** ^2^*	***F***	β	***t***
School adjustment	Authentic acting IM	0.279	57.234^***^	1.220	7.565^***^
Self-esteem	Authentic acting IM	0.173	20.924^***^	0.161	4.574^***^
School adjustment	Self-esteem	0.648	245.633^***^	2.793	20.010^***^
**Source of effect**	**Effect value**	**Boot SE**	**Boot LLCI**	**Boot ULCI**	**Relative utility value**
Total effect	1.220	0.161	0.903	1.536	
Direct effect	0.771	0.130	0.516	1.026	63%
Indirect effect	0.449	0.115	0.228	0.675	37%

^***^P < 0.001.

Which was statistically significant (*p* < 0.001), with a standardized path coefficient a1 = 0.161, which was significant (*p* < 0.001), and with a 95% confidence interval [0.0920, 0.230] does not contain 0.

The regression equation for the path of IM style, self-esteem to school adjustment for authentic acting was:


$$\begin{array}{c} \text{Y}=\;4.911+0.771\text{X}+2.793\text{M}, \end{array}$$

Which was statistically significant (*p* < 0.001), with a standardized path coefficient b1 = 2.793, significant (*p* < 0.001), and 95% confidence intervals [2.520, 3.067] that did not contain 0. The standardized path coefficient *c1'*=0.771, significant (*p* < 0.001), 95% confidence interval [0.516, 1.026] does not contain 0.

From Table 2, we know that path regression model had a significant coefficient of the direct effect path from the authentic acting IM style to school adjustment was significant before self-esteem was added to the equation (β = 1.220, *SE* = 0.161, *p* < 0.001). The effect stemming from the path became smaller after self-esteem was added (β = 0.771, *SE* = 0.130, *p* < 0.01), indicating that self-esteem plays a partial mediating role between the two. Meanwhile, further calculations revealed that the indirect effect of authentic acting IM style on school adjustment through self-esteem was 0.449, contributing 36.8% to the effect, with a 95% confidence interval [0.288, 0.675] does not contain 0. Thus, the effect of the mediating effect was considered statistically significant. Hypothesis 1 was partially supported.

Second, the mediator model with moderation was tested. Using authentic acting IM style as the independent variable, school adjustment as the dependent variable, self-esteem as the mediator, and age as the moderator, Model 7 was used in PROCESS to further examine whether age moderated the first half of the path of the mediated model. The results, as shown in Table 3, the interaction between authentic acting IM style and age was significant in predicting school adjustment (β = 0.017, *t* = 0.007, 95%CI = [0.003, 0.031], *p* < 0.001), and the moderating effect test was (BootLLCI = 0.035, BootULCI = 0.895), which did not contain 0, indicating a significant moderating effect. Thus, the first half of the path of the authentic acting IM style → self-esteem → school adjustment mediation model was moderated by age.

**Table 3:** Regression test of the relationship between authentic acting IM and school adjustment with self-esteem as a mediator age as a moderator.

**Regression models**	**β**	**SE**	***t* value**	**LLCI**	**ULCI**	***R* ^2^**	***F* value**
Outcome: SA						0.420	245.633^***^
Predictors: AC	0.771	0.130	5.931^***^	0.516	1.026		
SE	27.924	1.396	20.010^***^	25.189	30.670		
Outcome: SE						0.038	8.897^***^
Predictors: Age	−0.437	0.184	−2.374^*^	−0.800	−0.076		
SC	−0.008	0.011	−0.720^***^	−0.029	−0.013		
SA × Age	0.017	0.007	2.366^*^	0.003	0.031		

SA, school adjustment; AC, authentic acting IM style; SE, self-esteem.

^*^P < 0.05, ^***^P < 0.001.

**Figure 2:**
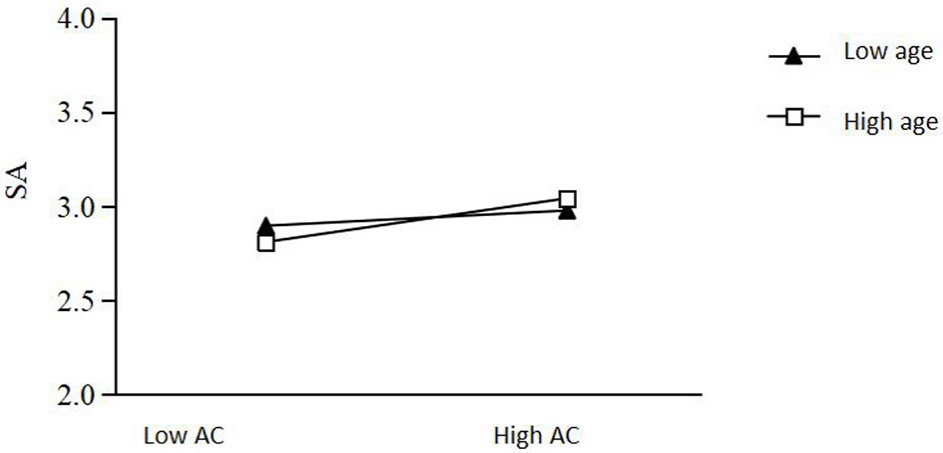
Moderating role of age between authentic acting IM style and school adjustment. Low AC: low score authentic acting, High AC: high score authentic acting.

Finally, to reveal more clearly the specific moderating role of age, a simple slope test was conducted to examine how the relationship between authentic acting IM style and self-esteem differed across age levels, and a simple effects analysis was plotted (e.g., Figure 2). The results of the test showed that authentic acting IM style was significantly less predictive of self-esteem for students in the lower age group (β = 0.009, *t* = 1.960, 95% CI = [0.000, 0.018], *p* < 0.001) than in the higher age group (β = 0.026, *t* = 4.772, 95% CI = [0.015, 0.036], *p* < 0.001). Age played a moderating role in the relationship between authentic acting IM style and self-esteem, and authentic acting IM style had a greater impact on self-esteem of students in the high age group.

#### The mediating role of self-esteem in the role acting IM style and school adjustment

**Figure 3:**
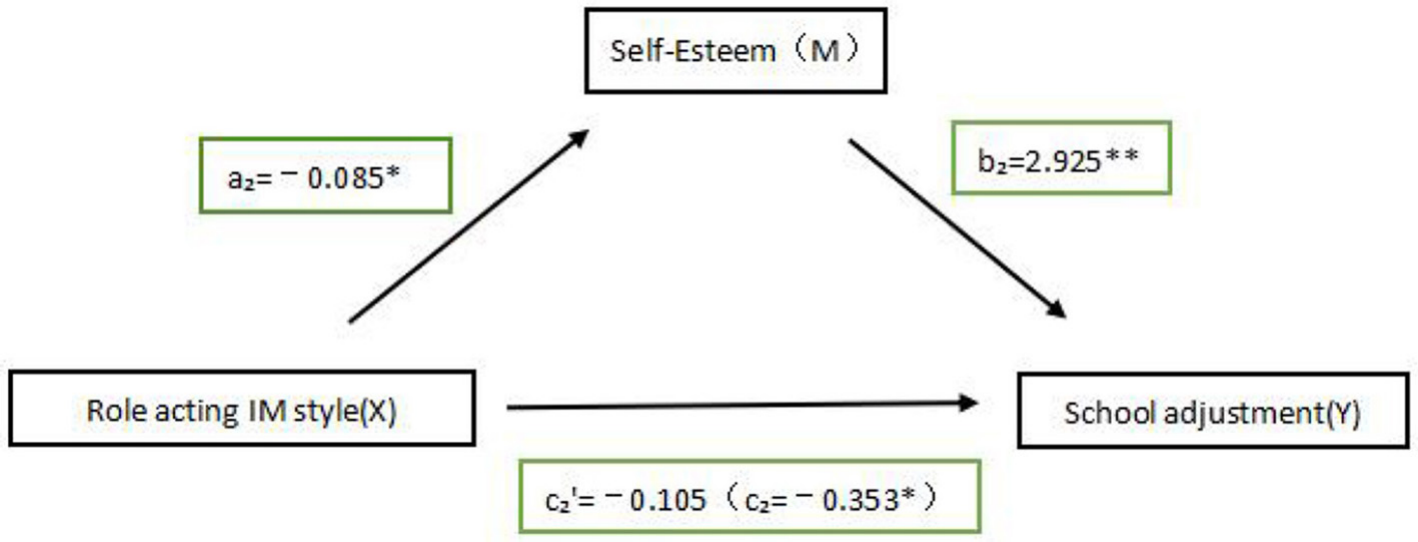
The mediating role of self-esteem in the role acting IM style and school adjustment. ^*^P < 0.05, ^**^P < 0.01.

From Table 4, in the model, *r*^2^ is 0.625, indicating that the model fits. As derived from Figure 3, the path regression equation for the path from role acting IM style to self-esteem was:


$$\begin{array}{c} \text{M}=\;31.614-0.085\text{X}, \end{array}$$

Which was statistically significant (*p* < 0.001), with a standardized path coefficient of a_2_ = −0.085 (*p* < 0.05), and 95% confidence intervals [−0.151, −0.019] does not contain 0.

**Table 4:** Intermediary effect detection, decomposition of total, direct, and indirect effects of self-esteem in role acting IM styles and school adjustment.

**Regression equation**		**Overall fit index**		**Significance of regression coefficients**	
**Outcome variables**	**Predictor variables**	***R** ^2^*	***F***	β	***t***
School adjustment	Role acting IM	0.085	4.987^*^	−0.353	−2.233^*^
Self-esteem	Role acting IM	0.009	6.379^*^	−0.085	−2.526^*^
School adjustment	Self-esteem	0.625	217.395^***^	2.925	20.656^***^
**Effect size**	**Effect value**	**Boot SE**	**Boot LLCI**	**Boot ULCI**	**Relative utility value**
Total effect	−0.353	0.158	−0.664	−0.043	
Direct effect	−0.105	0.125	−0.353	0.140	63%
Indirect effect	−0.248	0.114	−0.4712	−0.023	37%

^*^P < 0.05, ^***^P < 0.001.

The regression equation for the IM style, self-esteem to school adjustment path for role acting was:


$$\begin{array}{c} \text{Y}=23.493-0.105\text{X}+\;2.925\text{M}, \end{array}$$

Which was statistically significant (*p* < 0.001), with a standardized path coefficient of b_2_ = 2.925, significant (p < 0.001), and 95% confidence interval [2.647, 3.202] containing no zeros. The standardized path coefficient of c_2_' = −0.105, not significant (*p* = 0.399), 95% confidence interval [−0.350, 0.140] contains 0.

From Table 4, we know that the path regression model had a significant path coefficient for the direct effect of role acting IM style to school adjustment in the structural equation is significant before self-esteem is added in it (β = −0.353, *SE* = 0.158, *p* < 0.05), while the path of role acting IM style to school adjustment is insignificant after self-esteem is added in it (β = −0.105, *SE* = 0.125, *p* > 0.05), which indicates that self-esteem plays a fully mediating role between the two. Meanwhile, further calculations revealed that the indirect effect of role acting IM style to school adjustment through self-esteem was −0.248, contributing 70.2% to the effect, with a 95% confidence interval [−0.471, −0.023] does not contain 0. Thus, the mediating effect was considered to be statistically significant.

The moderating effect of age was further examined using Model 7, and the interaction between role acting IM style and age was not significant in predicting school adjustment (β = 0.042, *t* = 0.606, 95% CI = [−0.094, 0.178], *p* = 0.545), and the moderating effect test was (BootLLCI = −0.375, BootULCI = 0.617), contains 0, indicating that the moderating effect is not significant. Thus, the first half of the path of the role acting IM style → self-esteem → school adjustment mediation model was not moderated by age. Hypothesis 2 was partially supported.

## Discussion

The purpose of this study was to examine the relationship between students' impression management (IM) styles and school adjustment. Using a questionnaire-based approach, the findings indicated that different IM styles exert distinct effects on students' school adjustment. Specifically, self-esteem was found to mediate the relationship between IM styles and school adjustment. Further analysis revealed that self-esteem served as a partial mediator in the relationship between authentic acting IM style and school adjustment, and as a full mediator between role acting IM style and school adjustment. In addition, age was found to moderate the relationship between authentic acting IM style and self-esteem.

### Correlations between variables

#### Relationship between authentic acting IM style and self-esteem and school adjustment

The authentic acting impression management (IM) style is positively associated with self-esteem, suggesting that students who adopt this style are more likely to use positive coping strategies when facing academic and interpersonal challenges. Improvements in interpersonal relationships and academic performance can, in turn, enhance students' self-esteem (Lee, 2020). This IM style contributes to school adjustment because it involves expressing personal experiences, thoughts, feelings, needs, and desires that are believed to be true and consistent with the individual's authentic self (Harter, 2002; Schlenker and Weigold, 1992). By engaging in honest self-presentation, students make their situations more visible to teachers and peers, increasing the likelihood of receiving support and assistance (Kim and Lee, 2011). This not only strengthens peer relationships but can also positively influence teachers' perceptions, thereby enhancing academic outcomes and facilitating better adaptation to university life. Additionally, self-esteem is positively correlated with school adjustment, indicating that students with higher self-esteem are more capable of adapting to campus life by maintaining a positive outlook and effectively coping with academic, social, and emotional stressors.

#### Relationship between role acting IM style and self-esteem and school adjustment

The role acting impression management (IM) style is negatively associated with self-esteem, indicating that students using this strategy often cope with academic and interpersonal stress by concealing their true emotions and characteristics. When individuals rely heavily on such masking behaviors, their self-esteem may decline—especially if these behaviors are exposed or if stressors such as academic challenges and interpersonal tensions accumulate (Lee, 2020).

Role acting IM is also negatively related to school adjustment. Unlike authentic acting, which reflects one's true self, role acting is primarily aimed at fulfilling perceived social expectations in specific contexts (Safay, 2010). Individuals with a role acting IM style tend to present socially desirable images that do not align with their actual thoughts or emotions (Leary, 1995). This impression is maintained by pretending to have certain traits or needs, often involving the suppression of negative self-information. Although this may create a superficially positive image, it does not foster genuine social support. Once role acting is recognized by others, it can lead to feelings of distrust or deception, thereby damaging interpersonal relationships and negatively affecting academic performance (Safay, 2010).

As previously discussed, self-esteem is positively associated with school adjustment, as students with higher self-esteem are more likely to respond constructively to academic and social challenges.

### The mediating role of self-esteem

Self-esteem refers to an individual's evaluation of their own worth. It not only influences behavior but also shapes choices and coping strategies in social contexts. The analysis revealed that self-esteem partially mediates the relationship between authentic acting impression management (IM) style and school adjustment, while it fully mediates the relationship between role acting IM style and school adjustment. This suggests that IM styles not only have direct effects on positive or negative school adjustment but also exert indirect effects through self-esteem. These findings align with (Cheng and Furnham's, 2003) proposition that individuals can leverage their personality traits to mitigate adverse environmental influences and actively create more favorable environments for themselves.

For university students, the primary challenges within the school environment include academic stress, self-regulation, and interpersonal relationships (Zeng and Zhang, 2010). These factors can contribute to psychological and behavioral difficulties, ultimately undermining school adjustment. Faced with academic pressure, complex social interactions, and external expectations, students with low self-esteem tend to be more sensitive to stressors. They are more likely to experience frustration and often respond with avoidance or fantasy rather than confronting problems directly. Such tendencies may lead to academic procrastination, heightened anxiety and depression, and, in severe cases, hinder their ability to successfully complete their education (Schlenker and Weigold, 1992). In contrast, students with high self-esteem are better equipped to buffer external stressors. When confronted with similar challenges, they are more capable of transforming pressure into motivation, effectively managing their emotions, and addressing problems proactively. They are also more open to seeking support, accepting feedback from others, and mobilizing personal and social resources. These adaptive strategies enable them to focus more effectively on their academic responsibilities and daily life, thereby facilitating smoother adjustment to university life.

Students who adopt a role acting impression management (IM) style often lack self-confidence, leading to lower self-esteem compared to those who engage in authentic acting. This lack of self-esteem may result in a loss of self-identity and a tendency to mask or manipulate naturally occurring emotions. Self-esteem plays a crucial role in regulating psychological wellbeing and the quality of interpersonal relationships. Different IM approaches have distinct consequences, and authentic emotional expression is considered particularly valuable, as it has been shown to positively influence performance evaluations (Hochschild, 1983).

Role acting IM, characterized by the deliberate presentation of socially desirable but inauthentic images, may yield short-term benefits if executed effectively. However, empirical studies suggest that sustained deception increases the risk of detection, which can damage interpersonal trust, hinder academic performance, and ultimately lower self-esteem (Hafiz et al., 2020). This decline in self-esteem can directly impair students' school adjustment. These negative outcomes may also be influenced by the individual's IM competence (Safay, 2010). In contrast, students who adopt an authentic acting IM style align their internal states with their external expressions, avoiding the cognitive and emotional dissonance associated with deception. As a result, they are less likely to experience psychological distress and are better able to adapt to the school environment.

Therefore, for students who adopt an authentic acting impression management (IM) style, its influence on school adjustment is both direct and indirect through self-esteem. This suggests that interventions aimed at enhancing students' self-esteem and promoting authentic IM strategies can be effective in improving their overall school adjustment. In contrast, for students who rely on a role acting IM style, self-esteem serves as a key mediating factor. Consequently, improving these students' self-esteem may be a crucial pathway to enhancing their school adjustment, even if their IM style remains unchanged.

### The moderating role of age

The results indicated that age moderated the relationship between authentic acting impression management (IM) style and self-esteem, but not between role acting IM style and self-esteem. This may be explained by the developmental differences across age groups. As individuals grow older, they typically become more mature in social interactions and develop a clearer sense of self. Senior students tend to have a better understanding of their personal abilities and values, and are more likely to prioritize honesty and the building of trust. Their IM strategies thus lean toward authentic self-expression, which not only facilitates stronger interpersonal relationships but also enhances emotional satisfaction and perceived social support—factors that contribute positively to self-esteem. In contrast, younger students may still be in the process of exploring their identities, often experiencing confusion and uncertainty. As a result, they are more influenced by external expectations and social pressure, sometimes resorting to partially authentic or even inauthentic IM strategies in order to gain approval or avoid rejection. However, such divergence from the true self may result in internal alienation and self-discrepancy, ultimately leading to lower levels of self-esteem (Sun and Kang, 2020).

The moderating effect of age on the relationship between authentic acting impression management (IM) style and self-esteem can be attributed to the development of self-awareness and the accumulation of social experience. Senior students, with more mature self-concepts, are more likely to present their true selves, whereas junior students may rely more heavily on external recognition to shape their self-image. These differences not only influence the choice of IM style but also significantly impact the development of self-esteem.

Self-esteem is closely tied to psychological maturity. First-year university students often construct their self-worth based on external evaluations and social comparisons, rather than through honest self-expression. In contrast, older students may better understand the nuances of impression management, including when and how to authentically present themselves. Furthermore, differences in social skills, self-awareness, and accumulated successes between younger and older students contribute to distinct developmental trajectories (Gumz et al., 2020). As such, education and psychological support should intervene through moral, cognitive, and emotional dimensions to support students at different developmental stages.

Therefore, students who adopt authentic acting and role acting IM styles represent distinct groups requiring differentiated approaches to improve school adjustment. In guiding students toward developing self-esteem and managing impressions effectively, it is essential to consider individual differences—particularly age and the maturity of self-identity. By fostering a supportive environment, encouraging authentic self-expression, and cultivating emotional intelligence and social competence, educators can help students build healthy self-esteem and adapt more smoothly to university life.

## Limitations and future research

First, this study focused exclusively on university students from four comprehensive universities in China, without including participants from other educational institutions. However, different student populations—such as those in vocational colleges—may vary significantly in terms of gender distribution, academic ability, and self-esteem. For instance, while gender ratios in comprehensive universities are relatively balanced, vocational colleges often have a higher proportion of male students. Additionally, students in vocational colleges typically have lower academic achievement and potentially different levels of self-esteem, which may influence their use of impression management (IM) styles. These differences could alter the structural relationships explored in this study. Future research could benefit from comparing IM styles and their outcomes across different types of higher education institutions.

Second, cultural factors play a crucial role in shaping IM behaviors. Individuals from different cultural backgrounds display distinct IM styles and motivations in social, academic, and organizational settings. In Chinese culture, individuals tend to be more reserved and modest, often avoiding overt self-promotion and emphasizing behavioral restraint and adherence to social norms. This stands in contrast to Western cultural norms, where individuals are more likely to engage in assertive self-expression to demonstrate competence and build relationships, as described by Schlenker. While educational expectations related to academic success and school adjustment are common across cultures, the diverse political, economic, and educational contexts of different countries inevitably influence students‘ IM strategies. Furthermore, variations in educational systems may also shape students' school adjustment experiences. To improve the generalizability and applicability of current findings, future studies should consider replicating this research across broader cultural and institutional contexts.

Finally, the IM style scale used in this study, though psychometrically sound, primarily assessed verbal behaviors and did not account for non-verbal IM behaviors. As a result, some important aspects of students' impression management—such as body language, tone, or facial expressions—may have been overlooked. Moreover, while this study investigated how students' IM styles influence their school adjustment, it did not consider how these behaviors are perceived by others. Perceptions from teachers and peers can significantly impact students' academic and social outcomes. Therefore, future research should incorporate these external perceptions, as well as objective indicators such as academic grades or graduation success, to provide a more comprehensive understanding of the role of IM in school adjustment.

## Data Availability

The original contributions presented in the study are included in the article/supplementary material, further inquiries can be directed to the corresponding author.
